# Motion‐Compensated Diffusion Imaging With Phase‐Contrast for Robust Quantification of Regional Cerebral Blood Flow

**DOI:** 10.1002/mrm.70324

**Published:** 2026-03-02

**Authors:** Naoki Ohno, Tosiaki Miyati, Genki Nambu, Yu Ueda, Yuki Makino, Noam Alperin, Satoshi Kobayashi

**Affiliations:** ^1^ Faculty of Health Sciences, Institute of Medical, Pharmaceutical and Health Sciences, Kanazawa University Kanazawa Japan; ^2^ Graduate School of Medical Sciences, Kanazawa University Kanazawa Japan; ^3^ Department of Radiology University of Fukui Fukui Japan; ^4^ Philips Japan Tokyo Japan; ^5^ Radiology Division Kanazawa University Hospital Kanazawa Japan; ^6^ Department of Radiology University of Miami Miami Florida USA; ^7^ Department of Radiology Kanazawa University Hospital Kanazawa Japan

**Keywords:** diffusion‐weighted imaging, intravoxel incoherent motion, motion compensation, phase‐contrast, regional cerebral blood flow

## Abstract

**Purpose:**

To develop and evaluate a motion‐compensated diffusion imaging with phase‐contrast (MC‐DIP) technique for mitigating errors in regional cerebral blood flow (rCBF) quantification caused by physiological brain motion.

**Methods:**

Diffusion‐weighted images were acquired in 11 healthy volunteers on a 3.0 T MRI system using three gradient schemes: second‐order motion‐compensated (2nd‐MC), first‐order motion‐compensated (1st‐MC), and non‐compensated (non‐MC) diffusion gradients. Absolute rCBF maps were generated for each scheme by calibrating intravoxel incoherent motion‐derived relative perfusion maps with total cerebral blood flow measured by phase‐contrast MRI. The rCBF values from the DIP methods were compared in gray and white matter with a reference arterial spin labeling (ASL) measurement.

**Results:**

Both motion‐compensated schemes (2nd‐MC and 1st‐MC) provided significantly better biexponential fitting accuracy in gray matter compared with the non‐MC scheme (*p* < 0.05). In white matter, however, only the 2nd‐MC scheme resulted in a significant improvement over the other methods (*p* < 0.05). While rCBF values from all three DIP methods showed a strong positive correlation with ASL in gray matter (*ρ* ≥ 0.82, *p* < 0.05), only the 2nd‐MC‐DIP method demonstrated a significant positive correlation in white matter (*ρ* = 0.69, *p* < 0.05).

**Conclusion:**

The implementation of second‐order motion compensation within the DIP framework improves fitting accuracy, enabling robust rCBF quantification.

## 1 Introduction

The quantitative evaluation of regional cerebral blood flow (rCBF) is of significant clinical importance in managing various neurological conditions, particularly acute cerebral ischemia [1]. Established methods for rCBF measurement include arterial spin labeling (ASL) and dynamic susceptibility contrast (DSC) MRI; however, each has drawbacks, such as the dependence on arterial transit time in ASL and the requirement of contrast agents for DSC‐MRI [2, 3]. As an alternative, intravoxel incoherent motion (IVIM) analysis of diffusion‐weighted images provides an assessment of tissue microcirculation without using exogenous contrast agents [4]. A principal limitation of the IVIM approach, however, is that its perfusion‐related parameters are only semiquantitative, which makes absolute rCBF determination challenging.

To address the challenge of absolute quantification, diffusion imaging with phase‐contrast (DIP) was developed [5]. This technique enables the conversion of IVIM‐derived semiquantitative perfusion parameters into absolute rCBF values by normalizing them with total cerebral blood flow (tCBF) measured by phase‐contrast MRI. This approach is advantageous as it avoids contrast agents and allows for simultaneous acquisition of perfusion and diffusion information. Nevertheless, a challenge remains due to the inherent motion sensitivity of DWI. Specifically, bulk motion (i.e., physiological brain pulsation) can introduce signal artifacts that lead to an overestimation of diffusion coefficients [6]. Consequently, this may lead to a bias of DIP‐derived rCBF values.

To address the bulk motion‐induced errors in the DIP method, we developed and evaluated a motion‐compensated DIP (MC‐DIP) technique. We hypothesized that motion‐compensated diffusion gradients [7] would mitigate bulk motion effects, thereby enabling more reliable rCBF quantification compared with non‐compensated diffusion gradients. The primary objectives of this study were therefore: (1) to establish a robust framework for absolute rCBF quantification by mitigating motion‐induced artifacts; (2) to validate the reliability of the MC‐DIP method for quantifying rCBF against a reference ASL; and (3) to clarify the nature of physiological brain pulsation by comparing different orders of motion compensation (second‐order and first‐order motion‐compensated, and non‐compensated diffusion gradients).

## 2 Methods

**FIGURE 1:**
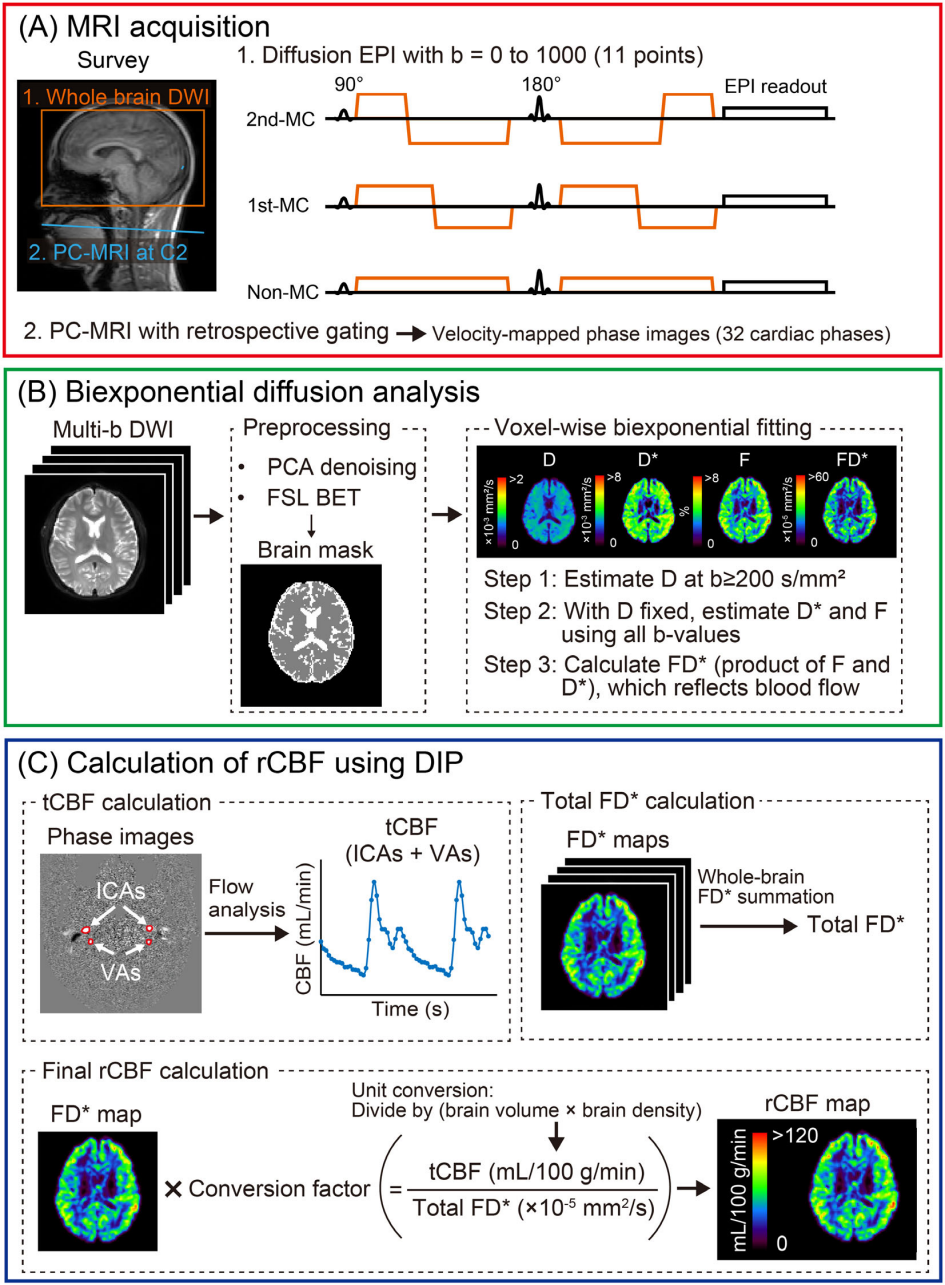
Schematic diagram of the analytical procedure for the motion‐compensated diffusion imaging with phase‐contrast (MC‐DIP). (A) Data acquisition: Diffusion‐weighted imaging (DWI) with multiple *b*‐values is acquired using three gradient schemes (2nd‐MC, 1st‐MC, and non‐MC), along with phase‐contrast (PC) MRI of the internal carotid arteries (ICAs) and vertebral arteries (VAs). (B) Biexponential diffusion analysis: The DWI data are processed using a stepwise biexponential fitting to estimate the true diffusion coefficient (*D*), perfusion‐related diffusion coefficient (D*), and perfusion fraction (*F*), from which a relative perfusion map (FD*) is calculated. (C) Absolute regional cerebral blood flow (rCBF) calculation: The PC‐MRI data are used to calculate total cerebral blood flow (tCBF). A conversion factor is determined by dividing tCBF by the whole‐brain sum of FD* values. The final absolute rCBF map is then generated by multiplying the FD* map by this conversion factor.

An overview of the analytical procedure for the MC‐DIP, from image acquisition to the final rCBF calculation, is schematically illustrated in Figure 1.

### 2.1 Subjects

The Institutional Review Board approved this prospective study (No. 848‐1), and written informed consent was obtained from all participants prior to MRI scan. Eleven healthy volunteers (9 men, 2 women; mean age, 23.9 ± 3.7 years) participated in this study. Inclusion criteria were as follows: age between 20 and 35 years, no history of neurological disorders or head trauma, and no contraindications to MRI.

### 2.2 MRI Acquisition

All MRI scans were performed using a 3.0 T MRI system (Ingenia, Philips Healthcare, Best, The Netherlands) equipped with a 32‐channel phased‐array head coil.

Single‐shot diffusion echo‐planar imaging (EPI) of the brain was conducted using second‐order motion‐compensated (2nd‐MC), first‐order motion‐compensated (1st‐MC), and motion‐uncompensated (non‐MC) diffusion gradient schemes (Figure 1A). Imaging parameters were as follows: repetition time (TR) = 4600 ms; echo time (TE) = 143 ms; flip angle = 90°; slice thickness/gap = 6 mm/0 mm; number of slices = 22; imaging matrix = 128 × 128; field of view (FOV) = 256 mm; sensitivity encoding (SENSE) acceleration factor = 2.1; multiband factor = 2; half‐scan factor = 0.7; number of signals averaged (NSA) = 2; and *b*‐values = 0, 10, 20, 30, 50, 100, 200, 400, 600, 800, and 1000 s/mm [2]. Diffusion gradients were applied along three orthogonal directions, and trace images were calculated for subsequent analysis. The gradient separation (Δ) and duration (δ) were 105.7/16.9 ms for 2nd‐MC, 26.0/25.1 ms for 1st‐MC, and 71.6/51.1 ms for non‐MC, resulting in different effective diffusion times across schemes. The acquisition time for each diffusion protocol was 5 min and 8 s.

To obtain tCBF, two‐dimensional PC‐MRI with retrospective peripheral gating was performed. The transverse imaging plane was placed at the mid‐C2 vertebral level (Figure 1A), perpendicular to the internal carotid arteries (ICAs) and vertebral arteries (VAs). Imaging parameters were TR = 10 ms; TE = 2.7 ms; flip angle = 25°; slice thickness = 5 mm; imaging matrix = 256 × 180; FOV = 150 mm; NSA = 1; SENSE factor = 2; velocity encoding = 80 cm/s. The acquisition time for PC‐MRI was approximately 1 min 30 s, depending on the subject's heart rate.

Reference rCBF measurements were performed using three‐dimensional gradient and spin‐echo (3D‐GRASE) pulsed continuous arterial spin labeling (pCASL). Imaging parameters were TR = 4638 ms; TE = 13 ms; flip angle = 90°; slice thickness = 6 mm; imaging matrix = 64 × 45; FOV = 240 mm; NSA = 2; labeling duration = 1800 ms; and post‐labeling delay (PLD) = 1525 ms. The acquisition time for ASL was 3 min and 17 s.

Structural imaging was performed using a 3D *T*_1_‐weighted fast field echo sequence with the following parameters: TR = 8.5 ms; TE = 4 ms; flip angle = 8°; slice thickness = 1 mm; imaging matrix = 288 × 216; FOV = 256 mm; compressed SENSE factor = 4; NSA = 1. The acquisition time for structural imaging was 2 min 20 s.

### 2.3 Biexponential Diffusion Analysis

For each of the three diffusion gradient schemes, voxel‐wise estimation of IVIM parameters was performed using biexponential fitting of the diffusion‐weighted signal decay (Figure 1B). Although previous studies employed a triexponential model [5, 8], the biexponential model was chosen because the extended TE for motion compensation reduces signal‐to‐noise ratio (SNR), making robust six‐parameter triexponential fitting challenging. Prior to parameter estimation, brain masks were generated from the non‐diffusion‐weighted (*b* = 0 s/mm^2^) images using brain extraction tools (BET; FSL, Oxford, UK) [9], ensuring that analysis was restricted to brain tissue and excluded areas like cerebrospinal fluid (CSF). Total brain volume was determined by the summation of voxel volumes within the *b*_0_ brain masks. Diffusion‐weighted images were subsequently denoised using a principal component analysis‐based algorithm [10] to improve parameter estimation.

The diffusion‐weighted signal (S) at different *b*‐values was fitted using a biexponential model described as follows:

(1) $$S=S_{0}\left[F\cdot \exp\left(-b\cdot D^{*}\right)+\left(1-F\right)\cdot \exp\left(-b\cdot D\right)\right],$$

where *S*_0_ is the signal intensity at *b* = 0 s/mm^2^, *D* ^*^ is the perfusion‐related diffusion coefficient, *D* is the true diffusion coefficient, and *F* is the perfusion fraction representing the contribution of blood perfusion to the diffusion‐weighted signal.

The fitting procedure was performed using a stepwise approach to improve robustness. Initially, the *D* was estimated using diffusion‐weighted images with high *b*‐values (≥ 200 s/mm^2^), assuming minimal contribution from perfusion at these *b*‐values. Subsequently, *D* ^*^ and *F* were estimated using all acquired *b*‐values with the fixed *D* values obtained from the first step. This process generated IVIM parametric maps, including a map of the product of *F* and *D* ^*^ (*FD* ^*^), which serves as a surrogate measure of relative blood flow [4]. All fitting procedures were performed using custom MATLAB scripts (MathWorks, Natick, MA, USA) employing a trust‐region algorithm. The normalized root mean squared error (nRMSE) was calculated to assess the goodness‐of‐fit of the biexponential model for each diffusion gradient scheme [11]. Lower nRMSE values indicate better fitting accuracy.

### 2.4 Calculation of rCBF Using DIP

An absolute rCBF map was derived for each diffusion dataset (2nd‐MC, 1st‐MC, and non‐MC) by calibrating the corresponding *FD* ^*^ map with tCBF measured by PC‐MRI (Figure 1C). Velocity‐mapped phase images were processed using the pulsatility‐based segmentation method [12]. The volumetric blood flow rate through the bilateral internal carotid arteries (ICAs) and vertebral arteries (VAs) was quantified by delineating the vessel lumen boundaries on velocity‐mapped phase images at the mid‐C2 vertebral level. The sum of the volumetric flow rates through these arteries represented the tCBF.

The tCBF values were converted to units of mL/100 g/min by dividing them by the product of the total brain volume and the brain tissue density (1.06 g/mL) [13]. Subsequently, the perfusion‐related diffusion parameters (specifically, the product of *F* and *D* ^*^, denoted as *FD* ^*^) were used to calculate voxel‐wise absolute rCBF values. Assuming a linear relationship between the FD* and rCBF [4, 14], we calculated a conversion factor as the ratio of tCBF to the total FD*, defined as the sum of FD* values across all voxels within the brain. Finally, rCBF maps in absolute units (mL/100 g/min) were generated by multiplying the *FD* ^*^ maps by this conversion factor.

### 2.5 Calculation of Reference rCBF Using ASL

Reference rCBF maps were calculated from the pCASL data based on a standard kinetic model [15]. Voxel‐wise rCBF was quantified using Equation (2).

(2) $$r{\mathrm{CBF}}_{\mathrm{ASL}}=\frac{6000\cdot \lambda \cdot \exp\left(\mathrm{PLD}/T_{1,\text{blood}}\right)}{2\cdot \alpha \cdot T_{1,\text{blood}}\left[1-\exp\left(-\tau /T_{1,\text{blood}}\right)\right]}\cdot \frac{\left(S_{\text{control}}-S_{\text{label}}\right)}{S_{\mathrm{PD}}},$$

where *λ* is the brain–blood partition coefficient (0.9 mL/g), *T*_1,blood_ is the longitudinal relaxation time of arterial blood (1650 ms), PLD is the post‐labeling delay (1525 ms), τ is the label duration (1800 ms), α is the labeling efficiency (0.85), *S*_control_ and *S*_label_ are the signal intensities of the control and labeled images, respectively, and *S*_PD_ is the signal from a proton density‐weighted image. The resulting ASL‐derived rCBF (*rCBF*_ASL_) maps were co‐registered to the DIP datasets using a linear image registration tool (FLIRT, FSL, Oxford, UK).

### 2.6 Measurements of rCBF

Mean rCBF values for gray matter (GM) and white matter (WM) were measured from all calculated rCBF maps (2nd‐MC, 1st‐MC, non‐MC‐DIP, and ASL). The *T*_1_‐weighted anatomical images were segmented into GM and WM probability maps using SPM12. High‐confidence masks for GM and WM were created with a probability threshold of 0.9. These masks were then co‐registered to each rCBF map (all DIP and ASL datasets) using the FSL FLIRT tool. The mean rCBF values for each method were measured using regions of interest for GM and WM, which were defined by the registered masks. To quantitatively assess the similarity of spatial rCBF distributions between DIP and ASL, we calculated the Jensen‐Shannon divergence (JSD) [16].

### 2.7 Statistical Analyses

All statistical analyses were performed using IBM SPSS Statistics (version 25, IBM, Armonk, NY, USA). A *p* value of less than 0.05 was considered to indicate statistical significance for all tests. To assess differences in biexponential fitting accuracy and spatial distribution similarity, the Friedman's test was performed to compare the nRMSE and JSD among the three DIP methods (2nd‐MC, 1st‐MC, and non‐MC), followed by pairwise comparisons using Wilcoxon signed‐rank tests with Bonferroni correction to account for multiple comparisons. The relationship between the rCBF values obtained from each of the three DIP methods and the reference ASL method was assessed using Spearman's rank correlation coefficient (*ρ*). Additionally, the agreement between rCBF values from each DIP method and ASL was evaluated using Bland–Altman analysis.

## 3 Results

**FIGURE 2:**
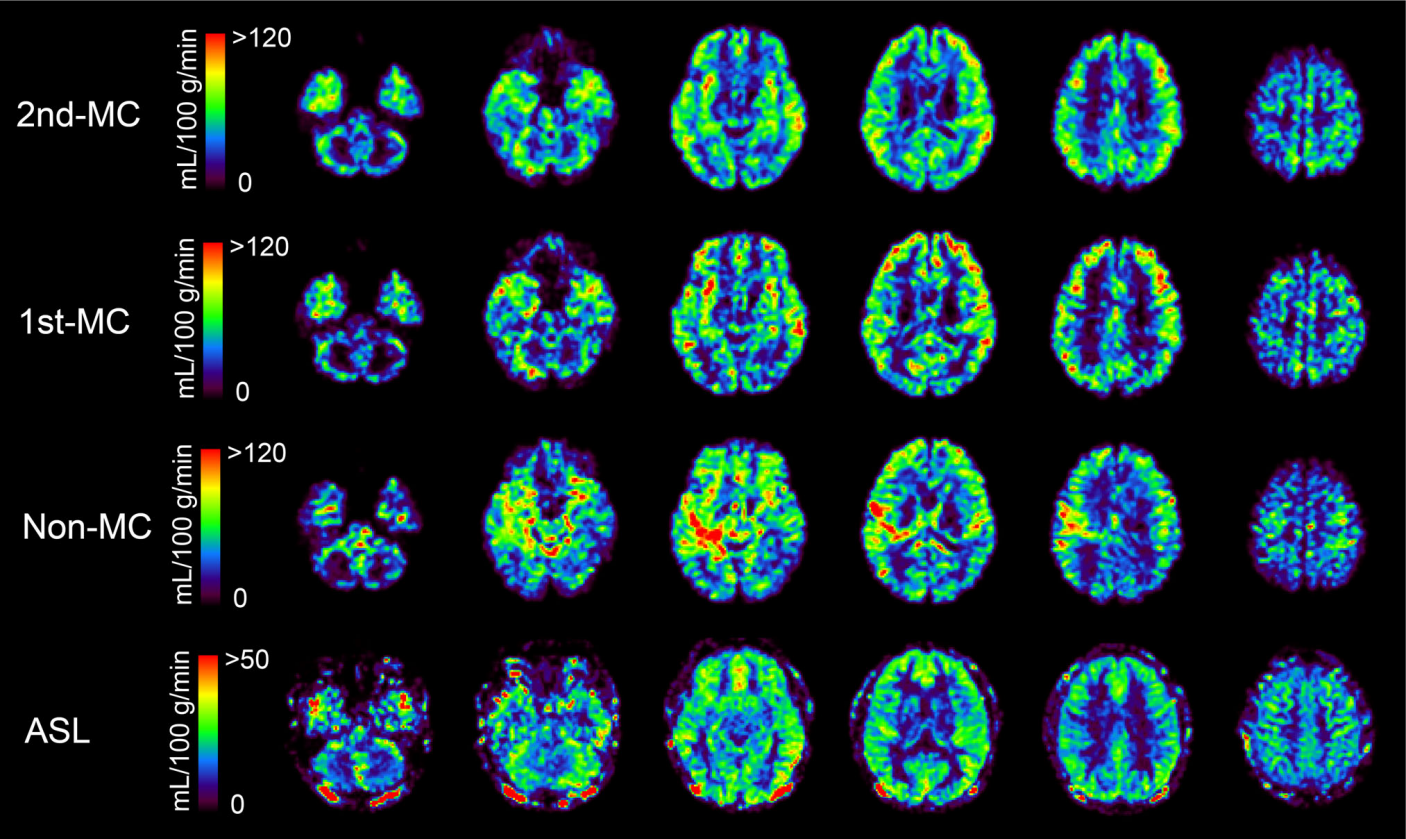
Representative rCBF maps from a single subject obtained using the three different DIP schemes and the reference ASL method. The non‐MC‐DIP shows prominent artifacts (areas of artificially high signal, red). These artifacts are partially reduced with 1st‐MC and most effectively suppressed with 2nd‐MC, resulting in maps with spatial distribution and contrast comparable to the ASL reference. rCBF, regional cerebral blood flow; MC‐DIP, motion‐compensated diffusion imaging with phase‐contrast; ASL, arterial spin labeling.

Figure 2 provides representative rCBF maps from a single subject for each of the four methods (2nd‐MC‐DIP, 1st‐MC‐DIP, non‐MC‐DIP, and ASL). The non‐MC‐DIP method showed substantial artifacts, appearing as regions of artificially high rCBF. While the 1st‐MC‐DIP method showed improvement, the 2nd‐MC‐DIP method most effectively reduced these artifacts, resulting in rCBF maps with GM/WM contrast and spatial distribution comparable to the ASL‐derived rCBF maps.

**FIGURE 3:**
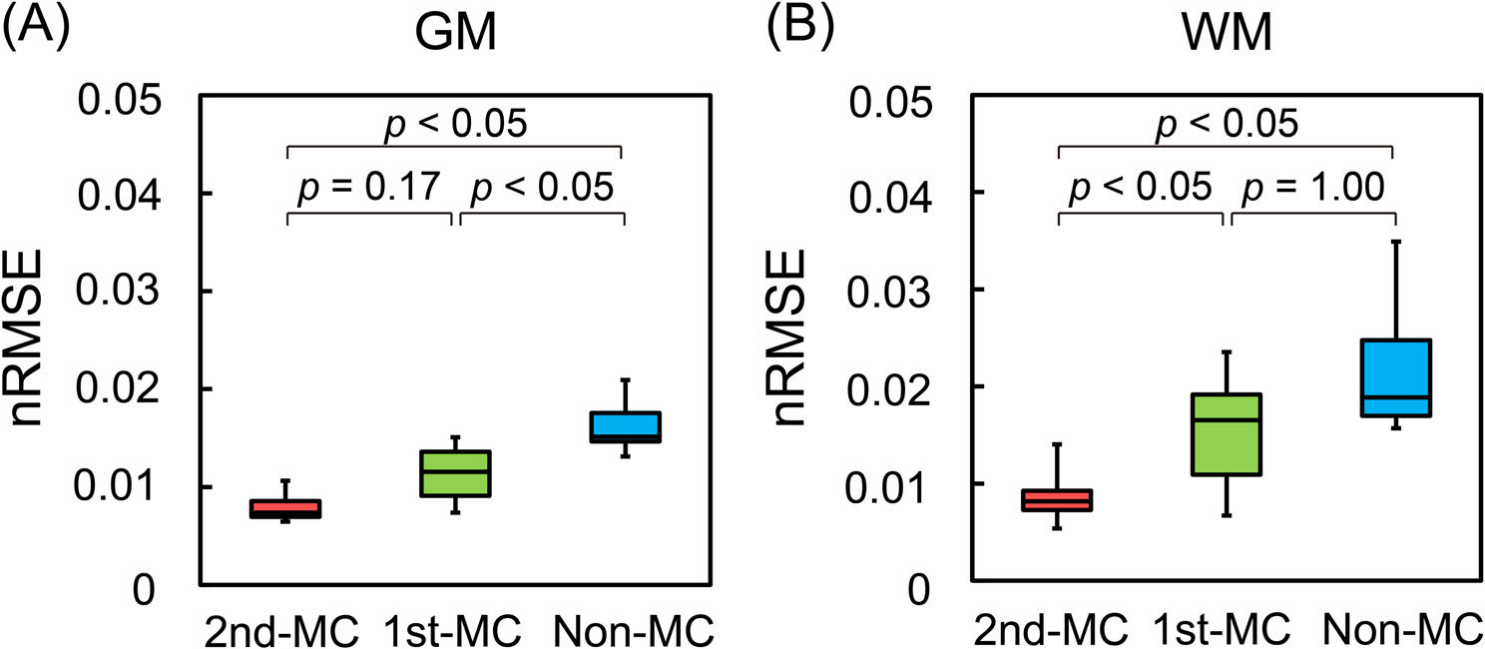
Comparison of biexponential fitting accuracy, as measured by the normalized root mean squared error (nRMSE), for (A) gray matter (GM) and (B) white matter (WM). The boxplots compare the second‐order motion‐compensated (2nd‐MC), first‐order motion‐compensated (1st‐MC), and non‐compensated (non‐MC) schemes. In GM, both the 1st‐MC and 2nd‐MC schemes were superior to the non‐MC scheme. In WM, however, only the 2nd‐MC scheme significantly reduced the fitting error compared with both other methods.

Figure 3 shows the quantitative comparison of fitting accuracy, as measured by nRMSE. For GM, both motion‐compensated methods (2nd‐MC and 1st‐MC) demonstrated significantly better fitting accuracy (lower nRMSE) than the non‐MC method (*p* < 0.05 for both). In WM, however, only the 2nd‐MC method provided a statistically significant improvement in nRMSE over both the 1st‐MC and non‐MC methods (*p* < 0.05 for both). Representative signal decay curves (Figure S1) showed substantial signal fluctuations at low *b*‐values for non‐MC, whereas 2nd‐MC demonstrated smooth biexponential decay.

**FIGURE 4:**
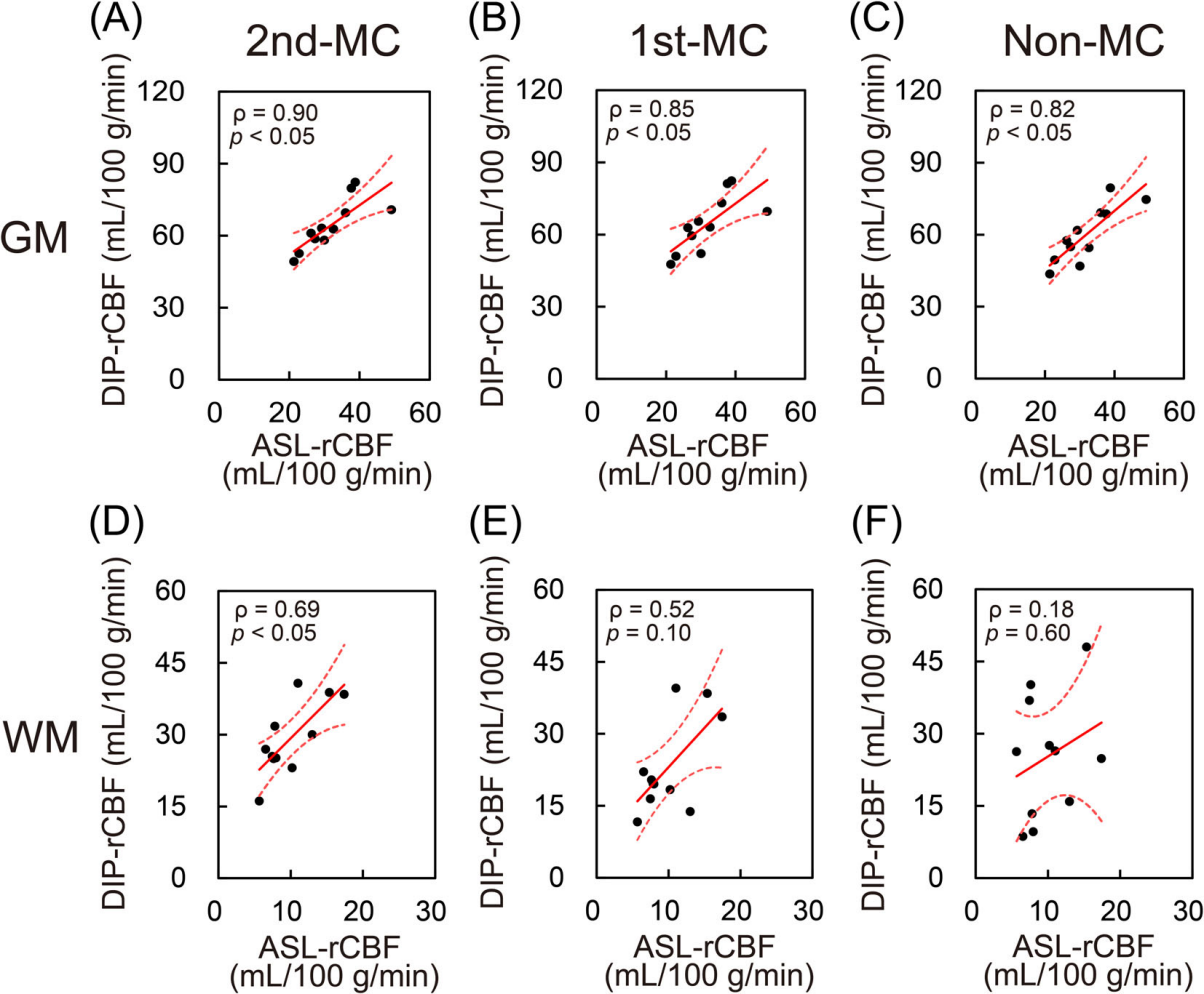
Scatter plots showing the correlation between DIP‐ and ASL‐derived rCBF in gray matter (GM; top row, A–C) and white matter (WM; bottom row, D–F). Each data point represents the mean rCBF value for each subject (*n* = 11). The plots correspond to the second‐order motion‐compensated (2nd‐MC; A, D), first‐order motion‐compensated (1st‐MC; B, E), and non‐compensated (non‐MC; C, F) schemes. Spearman's correlation coefficients (*ρ*) and *p* values are shown. While all schemes correlated with ASL in GM, only the 2nd‐MC scheme established a significant correlation in WM. DIP, diffusion imaging with phase‐contrast; ASL, arterial spin labeling; rCBF, regional cerebral blood flow.

Figure 4 illustrates the correlations between each DIP method and ASL. In GM, all three DIP methods showed a significant positive correlation with ASL: 2nd‐MC‐DIP (*ρ* = 0.90, *p* < 0.05), 1st‐MC‐DIP (*ρ* = 0.85, *p* < 0.05), and non‐MC‐DIP (*ρ* = 0.82, *p* < 0.05). In WM, however, only the 2nd‐MC‐DIP method demonstrated a significant positive correlation with ASL (*ρ* = 0.69, *p* < 0.05). The correlations for 1st‐MC‐DIP (*ρ* = 0.52, *p* = 0.10) and non‐MC‐DIP (*ρ* = 0.18, *p* = 0.60) in WM were not significant.

Bland–Altman analysis revealed a positive bias for all DIP methods relative to ASL in both GM and WM (Figure S2). In WM, 2nd‐MC‐DIP demonstrated the narrowest 95% limits of agreement, whereas non‐MC‐DIP showed substantially wider limits.

The similarity of spatial rCBF distributions was further assessed using JSD (Figure S3). In both GM and WM, 2nd‐MC‐DIP showed the lowest JSD, significantly different from non‐MC‐DIP (*p* < 0.05).

Table 1 presents the quantitative rCBF values. The mean rCBF values measured by the DIP methods in both GM and WM were comparable to the reference ranges reported in previous [^15^O]‐H_2_O PET studies [17, 18].

**TABLE 1:** Mean rCBF values (mL/100 g/min) for the three DIP methods and ASL in GM and WM.

		GM rCBF	WM rCBF
2nd‐MC‐DIP		64.4 ± 9.9	29.2 ± 7.3
1st‐MC‐DIP		64.3 ± 11.1	23.1 ± 9.2
Non‐MC‐DIP		60.1 ± 11.1	25.3 ± 12.2
ASL		31.9 ± 7.7	10.0 ± 3.6
[^15^O]‐H_2_O PET	Rostrup et al. [17]	61.0 ± 19.1	18.9 ± 5.9
	Ishii et al. [18]	50.4 ± 7.3	22.5 ± 3.1

*Note*: Literature values from previous [^15^O]‐H_2_O PET studies are included for reference. Values are mean ± standard deviation.

Abbreviations: ASL, arterial spin labeling; GM, gray matter; MC‐DIP, motion‐compensated diffusion imaging with phase‐contrast; rCBF, regional cerebral blood flow; WM, white matter.

## 4 Discussion

This study demonstrated that the implementation of 2nd‐MC within the DIP framework improves the robustness of rCBF quantification. Our results provide two major findings: first, the application of 2nd‐MC improved the accuracy of biexponential fitting in both gray and most notably white matter; and second, this improvement was essential for obtaining reliable white matter rCBF values that showed a strong correlation with ASL. These results fulfill the study's primary objective of overcoming the motion‐sensitivity limitations of the original DIP method, thereby establishing its feasibility for robust rCBF quantification.

Our study found that 2nd‐MC diffusion gradients substantially stabilized the biexponential fitting for IVIM analysis, especially in WM. This result can be explained by the inherent sensitivity of DWI to physiological brain motion, which corrupts the signal decay and leads to degraded model fitting [6]. The 2nd‐MC sequence mitigates this by using motion‐compensating gradients designed to be insensitive to acceleration, not just constant velocity [7, 19]. The superior performance of 2nd‐MC over 1st‐MC in our data therefore suggests that brain pulsation includes an acceleration component, which is inadequately corrected by velocity compensation alone. A recent work using high‐performance gradients has confirmed the superiority of 2nd‐MC, showing that it is more effective than 1st‐MC for stabilizing the signal phase against brain pulsation and can even eliminate signal dropout during brain motion [20]. This mechanism likely explains the pronounced improvement in WM, where deep‐tissue pulsatile motion is known to be a major confounding factor [6]. This improved fitting accuracy is a critical finding, as it provides the robust data quality necessary for the subsequent perfusion quantification to be considered reliable. Importantly, MC gradients selectively suppress coherent motion while preserving the IVIM perfusion signal. Bulk motion and large‐vessel flow produce refocusable phase shifts, whereas capillary flow produces irreversible phase dispersion manifesting as signal attenuation. Previous studies validated this principle, showing that flow‐compensated waveforms eliminate ballistic flow attenuation while preserving microcirculatory attenuation [21, 22]. The significant correlations between MC‐DIP and ASL‐derived rCBF confirm that perfusion signal is preserved despite motion compensation. Beyond bulk motion, MC encoding may also suppress macrovascular contamination. Coherent large‐vessel and CSF flow does not fit IVIM's microvascular assumption; MC gradients rephase these spins, filtering pseudo‐diffusion contributions. This may partly explain why 2nd‐MC showed the strongest correlation with ASL.

The second major finding of this study is that the improved fitting accuracy of the 2nd‐MC‐DIP method enabled reliable rCBF quantification in WM. The 2nd‐MC‐DIP was the only scheme to demonstrate a significant positive correlation with the ASL reference standard in WM. This finding was further supported by Bland–Altman analysis, which demonstrated that the 2nd‐MC‐DIP method had the narrowest limits of agreement with ASL in WM, indicating superior measurement consistency. The JSD analysis also supported this finding by demonstrating that 2nd‐MC‐DIP provided the highest spatial distribution similarity with ASL‐derived rCBF maps in both GM and WM. These results reflect a substantial improvement over our previous work, where the non‐motion‐compensated DIP technique failed to show any significant correlation with ASL in WM [5]. The lack of correlation in the previous study was attributed to methodological imprecision, where the low perfusion signal intrinsic to WM was likely obscured by motion‐induced artifacts [6, 8]. The present study confirms that by effectively suppressing motion‐related physiological noise, the 2nd‐MC scheme stabilizes the biexponential fitting sufficiently to detect the underlying physiological perfusion signal. This achievement is crucial because it expands the applicability of non‐contrast, diffusion‐based quantitative perfusion imaging to the entire brain parenchyma.

Visual inspection of the rCBF maps provided clear evidence for the necessity of higher‐order motion compensation. The non‐MC rCBF maps showed artifacts from physiological motion, appearing as areas of artificial hyperperfusion that could confound interpretation. While the 1st‐MC scheme, which corrects for constant velocity, visibly reduced these artifacts, regions of artificial hyperperfusion still remained. This persistence of artifacts strongly suggests that correcting for the velocity component alone is insufficient to address the complex nature of physiological brain motion. In contrast, the 2nd‐MC scheme, which also corrects for acceleration, effectively suppressed these residual artifacts, leading to cleaner maps with improved GM/WM contrast.

The mean rCBF values from all three DIP techniques were generally consistent with literature values from [^15^O]‐H_2_O PET [17, 18], which is considered the gold standard for rCBF quantification. This consistency supports the fundamental validity of our approach.

The demonstrated robustness of the 2nd‐MC‐DIP technique makes it a promising tool for clinical and research applications because of several advantages over other perfusion imaging methods. As a non‐invasive MRI technique, it requires no exogenous contrast agents, making it a safer alternative to DSC‐MRI, particularly for patients with renal impairment. Moreover, its perfusion measurement reflects the microvasculature and is therefore less susceptible to the effect of arterial transit time, a known challenge for ASL [2]. Most importantly, MC‐DIP provides both perfusion and diffusion information simultaneously. This unique capability is highly relevant for the assessment of ischemic stroke, as the combination of diffusion restriction (core) and perfusion deficits (penumbra) is critical for clinical decision‐making [23]. Taken together, these advantages suggest that MC‐DIP could play a valuable role in the clinical management of various neurological disorders.

This study demonstrated the feasibility of 2nd‐MC‐DIP for robust rCBF mapping. Nevertheless, several limitations should be acknowledged. First, the sample size was relatively small. Therefore, further studies with larger sample sizes, including varied ages and sexes, as well as clinical populations such as stroke or brain tumor patients, are required to validate its clinical utility. Second, ASL‐derived rCBF may be systematically underestimated due to 3D‐GRASE characteristics: diffusion sensitivity attenuating flowing spins [24], and *T*_2_ decay during the long echo train causing through‐plane blurring and GM‐WM partial volume effects [25]. However, these systematic effects are unlikely to substantially affect the DIP‐ASL correlation analysis. Third, we acknowledge the trade‐off between the longer TE required for motion compensation and a potential reduction in SNR. To ensure that any observed differences were attributable solely to the gradient design rather than SNR change, we fixed the TE for all DIP methods. However, given that the 2nd‐MC‐DIP showed a strong correlation in WM while our previous non‐compensated DIP with a shorter TE did not [5], the benefits of motion compensation appear to outweigh the penalty of an extended TE. Future work could investigate optimized 2nd‐MC gradient waveforms to shorten the TE [26]. Fourth, effective diffusion times differed across schemes due to motion‐compensated waveform constraints. Since IVIM‐derived perfusion parameters increase with diffusion time [22], spatially heterogeneous effects may persist locally, although tCBF calibration allows fair comparison of mean rCBF values. Fifth, although trace averaging provides robust estimates, rCBF consistency across diffusion‐encoding directions was not verified. Given reported D* anisotropy in WM [27], this may limit interpretation of WM rCBF. Sixth, the total acquisition time of approximately 7 min is long for routine clinical use. Optimizing the number and distribution of *b*‐values is a necessary next step to reduce scan time. Finally, simultaneous [^15^O]‐H_2_O PET validation using hybrid PET/MRI would be ideal to verify absolute quantitative accuracy and fully establish clinical utility.

## 5 Conclusions

This study demonstrated that the implementation of second‐order motion compensation within the DIP method improves the robustness of rCBF quantification. Our results provide two major findings. First, the motion‐compensated scheme, particularly the 2nd‐MC, substantially reduced the error in the biexponential signal fitting. Second, this improvement in fitting accuracy translated directly to more reliable perfusion measurements, as the 2nd‐MC‐DIP was the only method to yield white matter rCBF values that significantly correlated with the ASL reference standard. Therefore, 2nd‐MC‐DIP enables robust rCBF quantification.

## Funding

This work was supported by Japan Society for the Promotion of Science (22K07794).

## Conflicts of Interest

Yu Ueda is an employee of Philips. The motion‐compensated diffusion sequence used in this research was provided by Philips as part of a research agreement. All other authors declare that they have no conflicts of interest.

## Supporting information


**Figure S1:** Representative signal decay curves comparing the three diffusion gradient schemes in gray matter (GM; top row, A–C) and white matter (WM; bottom row, D–F). The plots correspond to the second‐order motion‐compensated (2nd‐MC; A, D), first‐order motion‐compensated (1st‐MC; B, E), and non‐compensated (non‐MC; C, F) schemes. Black circles represent measured signal intensities normalized to the *b* = 0 image (S/S_0_), and red solid lines indicate the biexponential fit. The normalized root mean squared error (nRMSE) is shown for each plot. Voxels were selected from a region showing high fitting error with the non‐MC scheme. The non‐MC scheme showed substantial signal fluctuations at low *b*‐values, whereas the 2nd‐MC scheme demonstrated smooth decay curves with improved fitting accuracy.
**Figure S2:** Bland–Altman plots showing the agreement between DIP‐ and ASL‐derived rCBF in gray matter (GM; top row, A–C) and white matter (WM; bottom row, D–F). The plots correspond to the second‐order motion‐compensated (2nd‐MC; A, D), first‐order motion‐compensated (1st‐MC; B, E), and non‐compensated (non‐MC; C, F) schemes. The solid red line represents the mean difference (bias), and the dashed red lines indicate the 95% limits of agreement (bias ±1.96 SD). All DIP methods showed a positive bias relative to ASL. In WM, the 2nd‐MC scheme demonstrated the narrowest limits of agreement. DIP, diffusion imaging with phase‐contrast; ASL, arterial spin labeling; rCBF, regional cerebral blood flow.
**Figure S3:** Comparison of spatial distribution similarity between DIP‐derived and ASL‐derived rCBF maps, as measured by the Jensen‐Shannon divergence (JSD), for (A) gray matter (GM) and (B) white matter (WM). The JSD is a symmetric measure derived from information theory that quantifies the similarity between two probability distributions, with values ranging from 0 (identical distributions) to 1 (completely different distributions). The boxplots compare the second‐order motion‐compensated (2nd‐MC), first‐order motion‐compensated (1st‐MC), and non‐compensated (non‐MC) schemes. In both GM and WM, the 2nd‐MC scheme showed the lowest JSD, with a significant difference compared with the non‐MC scheme.

## Data Availability

The data that support the findings of this study are available on request from the corresponding author. The data are not publicly available due to privacy or ethical restrictions.
