# Supplementary material for: Motion‐Compensated Diffusion Imaging With Phase‐Contrast for Robust Quantification of Regional Cerebral Blood Flow
Source: Magn Reson Med. 2026 Mar 2;96(1):238–46. doi: 10.1002/mrm.70324 (PMC13156429; doi:10.1002/mrm.70324)
Supplement: Supplementary file 1 — Figure S1: Representative signal decay curves comparing the three diffusion gradient schemes in gray matter (GM; top row, A–C) and white matter (WM; bottom row, D–F). The plots correspond to the second‐order motion‐compensated (2nd‐MC; A, D), first‐order motion‐compensated (1st‐MC; B, E), and non‐compensated (non‐MC; C, F) schemes. Black circles represent measured signal intensities normalized to the b = 0 image (S/S0), and red solid lines indicate the biexponential fit. The normalized root mean squared error (nRMSE) is shown for each plot. Voxels were selected from a region showing high fitting error with the non‐MC scheme. The non‐MC scheme showed substantial signal fluctuations at low b‐values, whereas the 2nd‐MC scheme demonstrated smooth decay curves with improved fitting accuracy. Figure S2: Bland–Altman plots showing the agreement between DIP‐ and ASL‐derived rCBF in gray matter (GM; top row, A–C) and white matter (WM; bottom row, D–F). The plots correspond to the second‐order motion‐compensated (2nd‐MC; A, D), first‐order motion‐compensated (1st‐MC; B, E), and non‐compensated (non‐MC; C, F) schemes. The solid red line represents the mean difference (bias), and the dashed red lines indicate the 95% limits of agreement (bias ±1.96 SD). All DIP methods showed a positive bias relative to ASL. In WM, the 2nd‐MC scheme demonstrated the narrowest limits of agreement. DIP, diffusion imaging with phase‐contrast; ASL, arterial spin labeling; rCBF, regional cerebral blood flow. Figure S3: Comparison of spatial distribution similarity between DIP‐derived and ASL‐derived rCBF maps, as measured by the Jensen‐Shannon divergence (JSD), for (A) gray matter (GM) and (B) white matter (WM). The JSD is a symmetric measure derived from information theory that quantifies the similarity between two probability distributions, with values ranging from 0 (identical distributions) to 1 (completely different distributions). The boxplots compare the second‐order motion‐compensated (2nd‐M [file MRM-96-238-s001.docx]

**Supporting Information**


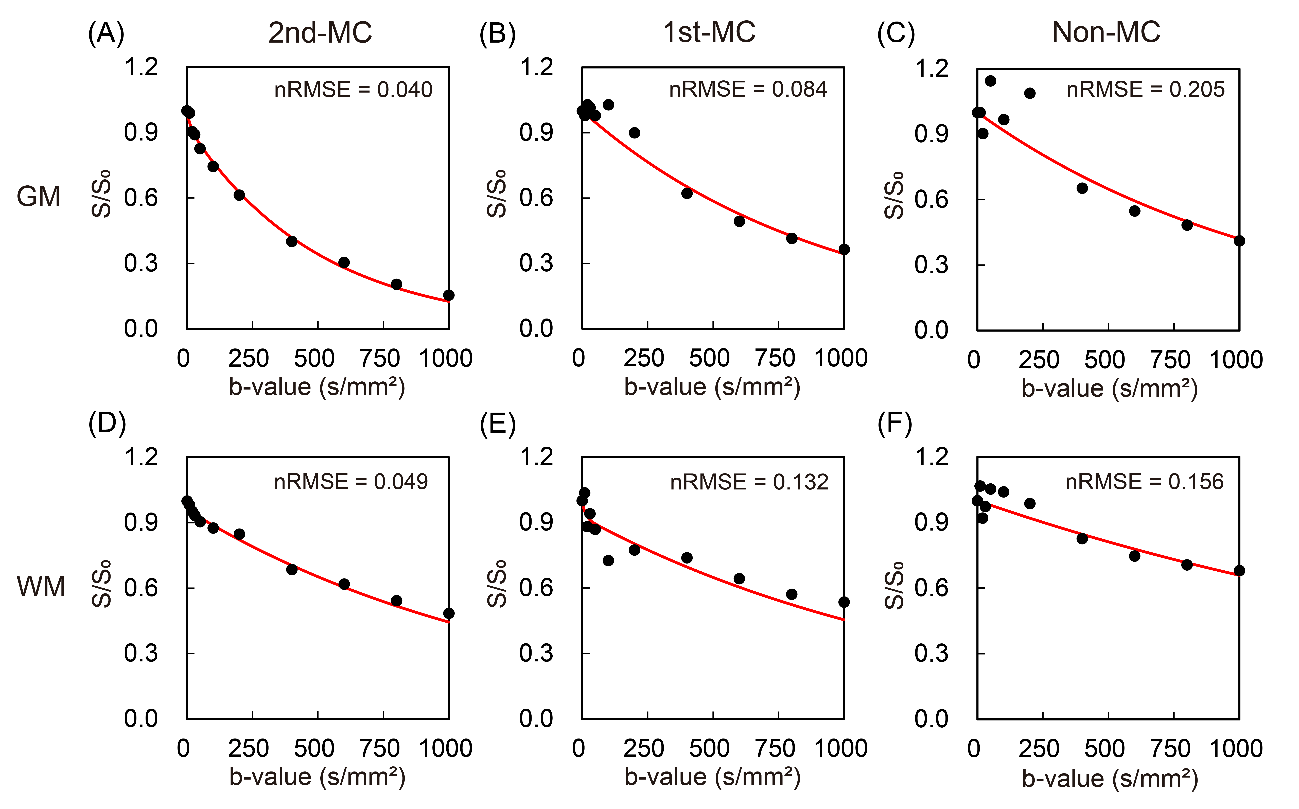


Figure S1. Representative signal decay curves comparing the three diffusion gradient schemes in gray matter (GM; top row, A–C) and white matter (WM; bottom row, D–F). The plots correspond to the second-order motion-compensated (2nd-MC; A, D), first-order motion-compensated (1st-MC; B, E), and non-compensated (non-MC; C, F) schemes. Black circles represent measured signal intensities normalized to the b = 0 image (S/S₀), and red solid lines indicate the biexponential fit. The normalized root mean squared error (nRMSE) is shown for each plot. Voxels were selected from a region showing high fitting error with the non-MC scheme. The non-MC scheme showed substantial signal fluctuations at low b-values, whereas the 2nd-MC scheme demonstrated smooth decay curves with improved fitting accuracy.


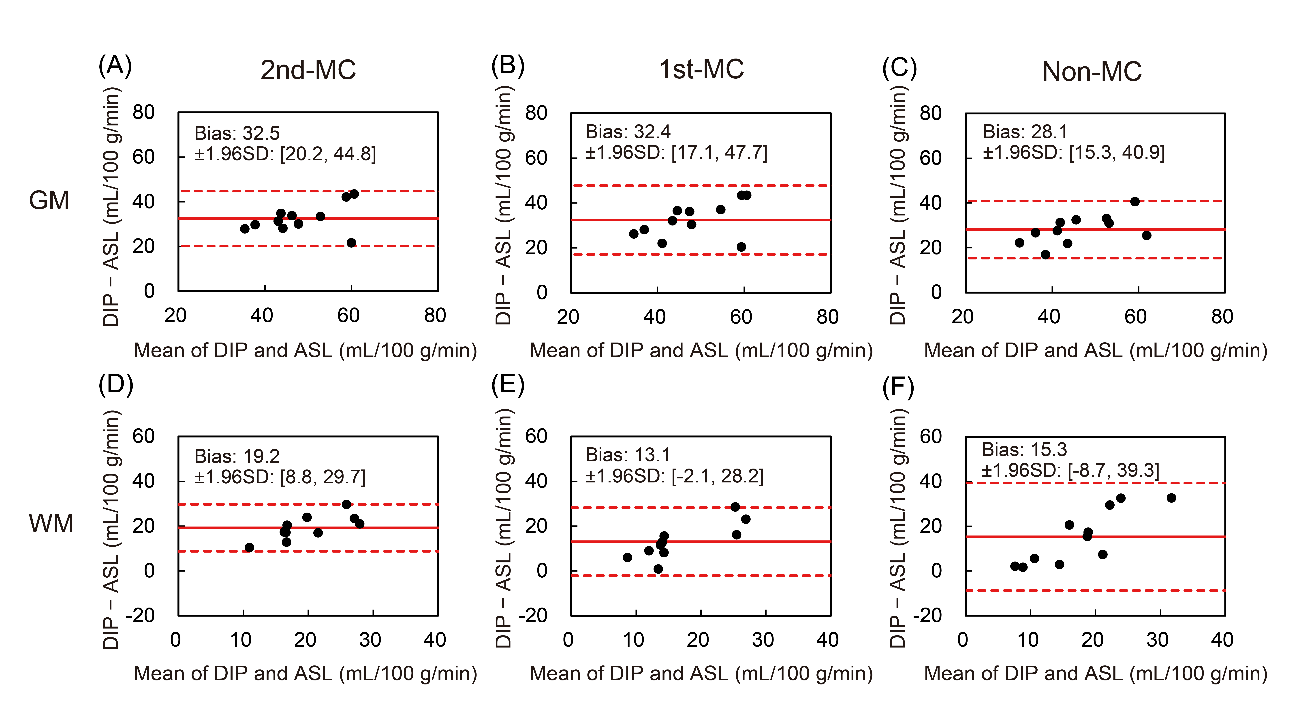


**Figure S2**. Bland-Altman plots showing the agreement between DIP- and ASL-derived rCBF in gray matter (GM; top row, A–C) and white matter (WM; bottom row, D–F). The plots correspond to the second-order motion-compensated (2nd-MC; A, D), first-order motion-compensated (1st-MC; B, E), and non-compensated (non-MC; C, F) schemes. The solid red line represents the mean difference (bias), and the dashed red lines indicate the 95% limits of agreement (bias ± 1.96 SD). All DIP methods showed a positive bias relative to ASL. In WM, the 2nd-MC scheme demonstrated the narrowest limits of agreement. DIP, diffusion imaging with phase-contrast; ASL, arterial spin labeling; rCBF, regional cerebral blood flow.


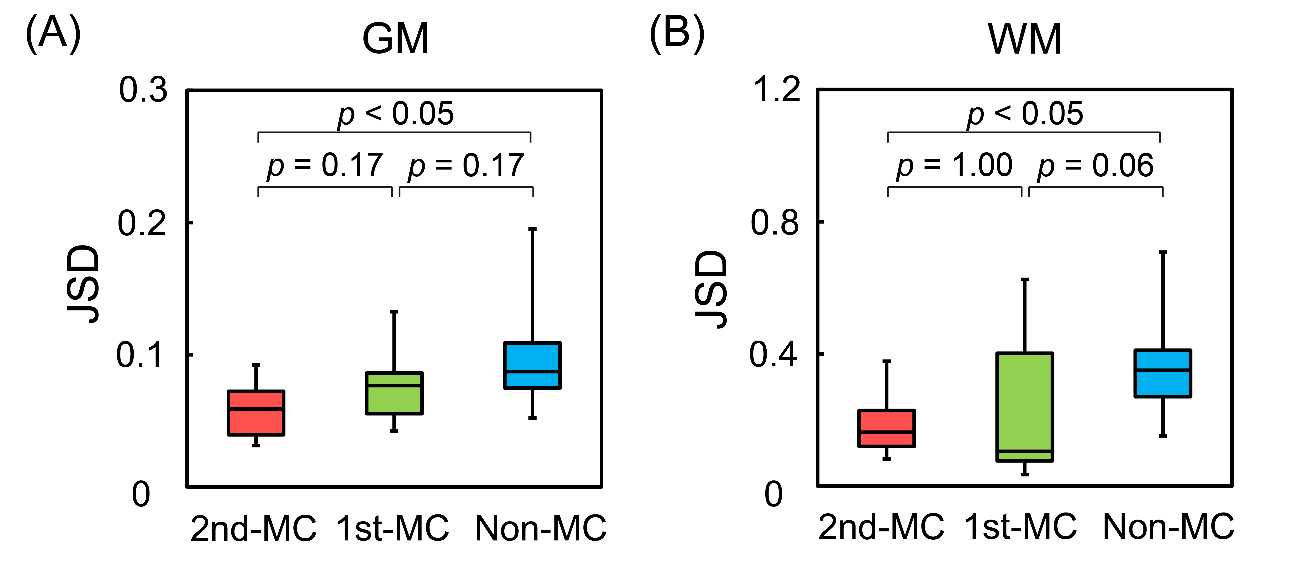


**Figure S3**. Comparison of spatial distribution similarity between DIP-derived and ASL-derived rCBF maps, as measured by the Jensen-Shannon divergence (JSD), for (A) gray matter (GM) and (B) white matter (WM). The JSD is a symmetric measure derived from information theory that quantifies the similarity between two probability distributions, with values ranging from 0 (identical distributions) to 1 (completely different distributions). The boxplots compare the second-order motion-compensated (2nd-MC), first-order motion-compensated (1st-MC), and non-compensated (non-MC) schemes. In both GM and WM, the 2nd-MC scheme showed the lowest JSD, with a significant difference compared with the non-MC scheme.
